# Leukocyte Dysregulation and Biochemical Alterations in End-Stage Kidney Disease Patients Under Hemodialysis

**DOI:** 10.3390/diseases13040090

**Published:** 2025-03-21

**Authors:** Gabriela Goyoneche Linares, Daysi Zulema Diaz-Obregón, Ana Granda Alacote, Michael Bryant Castro Núñez, María Gracia Castañeda Torrico, Alexis Germán Murillo Carrasco, Cesar Liendo Liendo, Katherine Susan Rufasto Goche, Víctor Arrunátegui Correa, Joel de León Delgado

**Affiliations:** 1 ONG Innovation and Science for the Care and Support of Society–INNOVACARE, Lima 15036, Peru; gabriela.goyoneche.l@gmail.com (G.G.L.); grandaalacote.ana@gmail.com (A.G.A.); magrita30890@hotmail.com (M.G.C.T.); 2 Health Technology Assessment and Research Institute (IETSI), EsSalud, Lima 15072, Peru; 3 Postgraduate School, Universidad Nacional Mayor de San Marcos, Lima 15011, Peru; dr.michael.castro@gmail.com; 4 Immunology and Cancer Research Group (IMMUCA), OMICS, Lima 15001, Peru; agmurilloc@usp.br; 5 Nephrology Center-CENESA, Lima 15072, Peru; cesarliendo2@gmail.com; 6 Postgraduate School, Universidad Nacional Federico Villarreal, Lima 15001, Peru; krufasto@unfv.edu.pe; 7 Faculty of Dentistry, Universidad Nacional Federico Villarreal, Lima 15001, Peru; 8 Faculty of Human Medicine, University of San Martín de Porres, Lima 15011, Peru; arruna9@hotmail.com; 9 Center of Virology Research, Faculty of Human Medicine, University of San Martín de Porres, Lima 15011, Peru; jdeleond@usmp.pe

**Keywords:** chronic kidney disease, diabetic nephropathy, hemodialysis, leukocytes, inflammation

## Abstract

Background: Patients with chronic kidney disease (CKD) exhibit changes in leukocyte dynamics, leading to altered hematological and biochemical parameters and deteriorating kidney function. In this study, we aim to investigate the correlation between leukocyte subpopulations and hematological and biochemical parameters in patients with end-stage CKD undergoing hemodialysis. Methods: This descriptive, analytical, cross-sectional study included 20 end-stage CKD patients on hemodialysis. Leukocyte subpopulations, including classical monocytes (CD14^++^/CD16^−^), intermediate monocytes (CD14^++^/CD16^+^), non-classical monocytes (CD14^+^/CD16^++^), CD4 T lymphocytes (CD3^+^/CD4^+^), CD8 T lymphocytes (CD3^+^/CD8^+^), B lymphocytes (CD3^−^/CD19^+^), NK cells (CD56^+^/CD16^+^), and iNKT cells (CD3^+^/CD56^+^), were analyzed using flow cytometry. Results: Patients with end-stage CKD on hemodialysis have decreased classical monocytes and increased non-classical monocytes frequency. A positive correlation was observed between non-classical monocytes and total lymphocytes (Rho-Spearman: R = 0.495, *p* = 0.027) as well as B lymphocytes (R = 0.567, *p* < 0.05). We discerned the immunological characteristics of diabetic kidney disease (DKD) and CKD due to other causes in this balanced cohort: B lymphocytes negatively correlate with alkaline phosphatase (R = −0.764, *p* < 0.05), parathyroid hormone (R = −0.929, *p* < 0.05), and ferritin (R = −0.893, *p* < 0.05). Additionally, in DKD, non-classical monocytes positively correlate with eosinophils (R = +0.691; *p* = 0.019) and classic monocytes with neutrophils (R = +0.627, *p* = 0.039). Meanwhile, a correlation between either total T lymphocytes or helper T lymphocytes and serum albumin was detected on patients with nephropathy due to other causes. Conclusions: CKD alters classical and non-classical monocyte frequency, whilst T and B lymphocyte frequency positively correlates to the proinflammatory non-classical monocytes. In DKD patients, the uremic environment increases classic monocytes, CD16+ inflammatory monocytes, neutrophils, eosinophils, and B lymphocytes. The described leukocyte dynamic correlates with alkaline phosphatase, parathyroid hormone, iron, and serum albumin serological concentration.

## 1. Introduction

Chronic kidney disease (CKD) affects approximately 10% to 14% of the general population [1]. The causes of CKD are diverse, including type 2 diabetes mellitus (DM2). Indeed, the primary cause of end-stage CKD is diabetes (diabetic kidney disease, DKD), when renal replacement therapies such as peritoneal dialysis, hemodialysis, or kidney transplantation become necessary [2]. In Peru, up to 25% of patients with DM2 develop DKD [3].

Inflammation is a prominent factor of DKD, and its progression leads to end-stage CKD [4]. Immune function is compromised by the retention of uremic toxins, which activate innate immune cells and lead to the continuous production of cytokines and reactive oxygen species, resulting in tissue damage [5]. Dysfunctions and a reduction in the number of lymphocytes have been observed, contributing to immunosuppression and an increased risk of infection in patients [6,7]. Additionally, endothelial dysfunction, vascular calcification, oxidative stress, and inflammation are exacerbated, collectively accelerating the progression and severity of nephropathy [8,9].

End-stage CKD induces senescent T cells and monocytes, with an increased expression of cytotoxic CD8 T lymphocytes and inflammatory-profile monocytes. Intermediate monocytes (CD14^++^/CD16^+^) and non-classical monocytes (CD14^+^CD16^++^) contribute to kidney dysfunction in diabetic patients [10,11]. Classical monocytes (CD14^++^CD16^−^) are associated with phagocytic function and the initial inflammatory response, characterized by the expression of IL-10 and low levels of TNF-α, while CD16^+^ monocytes (both non-classical and intermediate) are involved in inflammatory and infectious processes, expressing higher levels of pro-inflammatory molecules. Non-classical monocytes stimulate the production of TNF-α and IL-1β [12].

In patients undergoing hemodialysis, CD16^+^ monocyte subpopulations have shown signs of altered maturation due to dialysis or uremia [13]. Although studies have documented that dysregulation of monocyte maturation and function is associated with DKD and its complications, it is not well characterized [4]. These patients exhibit persistent and progressive dysregulation in leukocyte subpopulations, with an increased tendency toward monocyte expansion, cytokine release, and free radical production, which exacerbate oxidative stress and endothelial dysfunction [14]. This dysregulation leads to impaired monocyte function in phagocytosis and antigen presentation. Alterations in T and B lymphocyte function, significant reductions in CD4^+^ T lymphocytes, and low CD4^+^/CD8^+^ ratios (with variations in the CD8^+^ population) are linked to an increased risk of infections [15,16].

The variability of leukocyte subpopulations and their association with oxidative stress and inflammation earn significant interest, particularly concerning their prognostic value and its correlation with hematological and biochemical parameters [5,6]. Identifying risk factors for disease progression and novel monitoring strategies for patients with chronic-degenerative diseases, such as end-stage CKD, remain critical priorities. This study aims to determine the correlation between leukocyte subpopulations and hematological and biochemical parameters in CKD patients, either diabetic or not, undergoing hemodialysis.

## 2. Materials and Methods

### 2.1. Study Description

This descriptive, analytical, and cross-sectional study was conducted on a population of patients with end-stage CKD receiving outpatient hemodialysis at the Private Nephrological Center (CENESA S.A.). All participants were referred from the Edgardo Rebagliati Martins National Hospital and were admitted to CENESA with a diagnosis, treatment plan, and the need for outpatient hemodialysis.

This study was conducted in two phases. The first phase involved collecting sociodemographic and clinical data from patients’ medical records, along with the results of various biochemical parameter assessments. The second phase focused on collecting blood samples for hematological assays. This study was conducted in accordance with the protocol approved by the Institutional Research Ethics Committee of the Faculty of Human Medicine at the University of San Martin de Porres (Official Letter No. 0104-2024-CIEI-FMH-USMP).

### 2.2. Patients

Considering an estimated population of 2.48 million elderly individuals in Peru with CKD, of which approximately 1.75% are in advanced stages and opt for hemodialysis [17], there is a population of 43,000 Peruvian patients over the age of 55 with end-stage CKD in hemodialysis. Using this value and a confidence level of 80%, the OpenEpi program (openepi.com) indicates that a sample of 17 individuals is required to achieve a power of 90%. Twenty patients aimed to participate and signed their informed consent to be included in the study. The etiology of end-stage CKD was registered based on referral reports from the reference hospital, which included their diagnosis, comorbidities, current treatment, and clinical history. Patients with hematological, oncological, autoimmune diseases, or acute infections at the time of blood sample collection were excluded from this study. All patients underwent hemodialysis using the Helixone^®^ polysulfone membrane.

### 2.3. Sample Collection and Processing

Peripheral blood was collected in tubes with ethylenediaminetetraacetic acid (EDTA) (Becton-Dickinson, San José, CA, USA). To isolate peripheral blood mononuclear cells (PBMCs), blood samples were diluted in phosphate-buffered saline (1× PBS; 1:1 *v*:*v*), added to 3 mL of Ficoll-Paque PLUS (Histopaque^®^-1077, Sigma-Aldrich, St. Louis, MO, USA), and centrifuged at 2000 rpm (Thermo Scientific SL 8 Centrifuge, Thermo Fisher Scientific, Waltham, MA, USA), for 20 min, at room temperature. The PBMCs were then collected and washed twice with 1x PBS solution. PBMCs were counted in a Neubauer chamber.

### 2.4. Flow Cytometry

PBMCs (~6 × 10^6^ per patient) were resuspended in a PBS solution with 10% fetal bovine serum and 0.1% sodium azide (FACS solution, catalog number 349202, BD FACS^TM^). To perform an immunophenotypic analysis of cell subpopulations, 5 µL/million PBMCs of fluorophore-conjugated monoclonal antibodies were used. T lymphocyte detection: PE-Cy7-CD3, APC-CD4, PerCP-Cy5.5-CD8, PE-CD45RA, FITC-CCR7; B lymphocyte detection: PercP-Cy5.5-CD19; monocyte detection: PE-Cy7-CD3, FITC-CD14, APC-CD16; NK and NKT lymphocyte detection: APC-CD16, PE-CD56, PE-Cy7-CD3 (catalog IDs and suppliers are reported in Table 1). The cells were incubated with the antibodies for 30 min at 4 °C, then washed 3 times by centrifugation at 2000 rpm for 5 min, and finally resuspended in 200 µL of solution.

Differentiation of cell subpopulations was carried out in a FACSLyric^TM^ flow cytometer (BD Biosciences, Franklin Lakes, NJ, USA) using the Infinicyt™ analysis software v.2.0. Leukocyte subpopulations were detected based on the following markers: classical monocytes (CD14^++^/CD16^−^), intermediate monocytes (CD14^++^/CD16^+^), non-classical monocytes (CD14^+^/CD16^++^), T helper cells (CD3^+^/CD4^+^), cytotoxic T cells (CD3^+^/CD8^+^), B cells (CD3^−^/CD19^+^), NK cells (CD56^+^/CD16^+^), and iNKT cells (CD3^+^/CD56^+^). Unlabeled cells or cells stained with fluorophore-conjugated irrelevant antibodies were used as negative controls.

### 2.5. Statistical Analysis

A comparative analysis of qualitative variables was conducted using the Chi-square test or Fisher’s exact test. For numerical variables, Student’s *t*-test or the Mann–Whitney U test was used, depending on the data distribution (normal or non-normal), as determined by graphical methods and normality tests (Shapiro–Wilk test and analysis of skewness and kurtosis). The correlations between leukocyte subpopulations and hematological and biochemical parameters were analyzed using the Spearman test. A 95% confidence level was considered. All the analyses were performed using STATA v.15 software.

## 3. Results

### 3.1. Sociodemographic and Etiopathogenic Characteristics of the Patients

As shown in Table 2, the median age of end-stage CKD patients is 66.5 years. More than 50% of patients have long-lasting uremia, are diabetic and hypertensive, and have normal weight based on BMI.

### 3.2. Analysis of Hematological and Biochemical Parameters

Hematological and biochemical markers, determined after hemodialysis, evidence higher concentrations of phosphorus, CRP, creatinine, and urea than the reference values. However, reduced levels of hemoglobin, hematocrit, transferrin, and iron were detected (Table 3). Of note, Kt/v associated with urea excretion/retention almost duplicated the reference value.

### 3.3. Leukocyte Subpopulations in End-Stage CKD Patients

The quantification of leukocytes (Table 4) and monocytes (Table 5) were within the reference range for end-stage CKD patients. However, the evaluation of monocyte subpopulations evidences a decreased frequency of classical monocytes while the frequency of non-classical monocytes increased in comparison to the reference values.

### 3.4. Correlation of Leukocyte Subpopulations with Hematological and Biochemical Parameters

Considering the relevance of DM in end-stage CKD onset and progression, we explored whether leukocyte subpopulations have any correlation with hematological and biochemical parameters based on this comorbidity. In patients with DKD, a positive correlation was detected between total monocytes and CD4^+^ T cells, while B lymphocytes negatively correlate with alkaline phosphatase, parathyroid hormone, and ferritin. Additionally, negative correlations were detected between CD4^+^ T lymphocytes and parathyroid hormone and between iNKT cells and serum iron concentration (Table 6).

On the other hand, patients with nephropathy due to other causes have a significant positive correlation between T lymphocytes and CD4^+^ T cells with serum albumin concentration. A significant negative correlation exists between T lymphocytes or CD4^+^ T lymphocytes with neutrophils count (Table 6).

**Figure 1:**
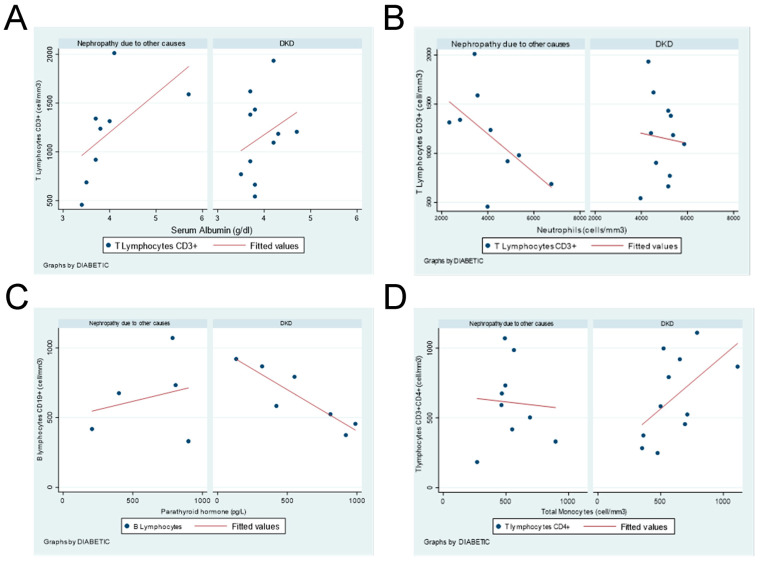
Correlation between leukocyte subpopulations and hematological parameters in DKD or nephropathy due to other causes in end-stage CKD patients. (**A**) CD3^+^ T Lymphocytes vs. serum albumin. (**B**) CD3^+^ T lymphocytes vs. neutrophils. (**C**) B lymphocytes vs. parathyroid hormone. (**D**) CD4^+^ T lymphocytes vs. total monocytes.

Unveiled correlations of T and B lymphocytes with serum albumin, parathyroid hormone, neutrophils, and monocytes are shown in Figure 1, either in DKD or no-diabetic end-stage CKD patients.

Table 7 shows that despite the ethology of end-stage CKD, non-classical monocytes significantly correlate with total lymphocytes and B lymphocytes. However, if DKD and nephropathy due to other causes in end-stage CKD patients are discerned, classical monocytes have a significant positive correlation with neutrophils, and the non-classical monocyte subpopulation significantly correlates to eosinophils. Otherwise, in non-diabetic patients, a positive correlation was detected between classical monocytes and neutrophils, as well as non-classical monocytes and total lymphocytes (Table 8).

## 4. Discussion

Detailed characterization of hematological and biochemical status in end-stage CKD patients is fundamental for disease prognosis and success treatment prediction. Evidence about statistical and biological correlation among these biomarkers is scarce, particularly considering that end-stage CKD etiology adds a layer of complexity to any evaluation. Regarding the relevance of DKD on progression to end-stage CKD, we explored the overall values and correlations among hematological and biochemical parameters on Peruvian patients under hemodialysis.

The end-stage CKD patients included in this study have increased values in phosphorus and CRP serological concentration. After hemodialysis, their creatinine and urea concentrations increase. Indeed, the urea excretion/retention Kt/v variable almost duplicated the reference value. Hemoglobin, hematocrit, transferrin, and iron are reduced irrespectively of the disease etiology. Regarding immunity cells, leukocytes are within the reference range, but the frequency of classical monocytes is lower, while the frequency of non-classical monocytes is higher than the reference values. Interestingly, in end-stage CKD patients, the amount of T and B lymphocytes correlates with serum albumin and parathyroid hormone concentration, as well as neutrophils and monocytes cells. Also, non-classical monocytes, total lymphocytes, and B lymphocytes are positively correlated.

In nephropathic diabetic end-stage CKD patients, helper CD4^+^ T cells positively correlate with total monocytes while negatively correlating with parathyroid hormone. B lymphocytes negatively correlate with alkaline phosphatase, parathyroid hormone, and ferritin. Additionally, classical monocytes correlate with neutrophils, while non-classical monocytes significantly correlate with eosinophils. Interestingly, the scarce iNKT cell population correlates with serum iron concentration.

When nephropathy due to other causes in end-stage CKD origin is considered, serum albumin concentration positively correlates with T lymphocytes and helper CD4^+^ T cells. However, neutrophil count negatively correlates with these lymphocytes. Otherwise, a positive correlation was detected between classical monocytes and neutrophils, as well as non-classical monocytes and total lymphocytes.

Despite the overall biochemical parameters showing no significant alterations in evaluated end-stage CKD patients, serum phosphorus concentration, alkaline phosphatase, and serum transferrin are slightly out of the reference ranges. The association between high phosphorus levels and the progression of DKD, cardiovascular calcification, and increased mortality have been described [18,19]. Alkaline phosphatase is a marker of secondary hyperparathyroidism in CKD, linked to vascular calcification and cardiovascular morbidity. The reduction in serum transferrin suggests a decreased capacity for iron transportation due to kidney damage [20].

Monocyte subpopulations include 80–90% of classical monocytes and 10–20% of CD16^+^ non-classical monocytes in healthy individuals. Our study shows an increase in non-classical monocytes and a decrease in classical monocytes in end-stage CKD patients, suggesting the progression of classical monocytes into non-classical forms, a process potentially moderated by hemodialysis [21,22,23,24,25]. Inflammatory monocytes increase with age, particularly in CKD patients, with classical monocytes decreasing and non-classical monocytes rising, a trend intensified by the uremic environment [26]. Classic monocytes are reduced in the overall patient population and correlates with neutrophils, either in diabetic or non-diabetic patients. In diabetic patients, CD16^+^ monocytes show increased pro-inflammatory activity, particularly in DKD [9,27]. Recent transcriptome and proteome studies highlight the functional differences between monocyte subpopulations, such as the role of CD14^+^CD16^+^ monocytes in gene expression regulation during phagocytosis and CD14^+^CD16^−^ monocytes in antimicrobial functions. Additionally, CD16^+^ Slan^+^ monocytes, elevated in kidney lesions, contribute to inflammation in conditions like lupus nephritis, underscoring the importance of further research into monocyte subpopulations and immunity [28,29].

The negative correlation between ferritin, parathyroid hormone, and alkaline phosphatase levels can be explained due to the role of these biomarkers in B cell function [30,31,32]. Parathyroid hormone binds to the CD3^+^CD4^+^ T lymphocyte receptor influencing helper T cells, a reasonable support for negative correlation between both variables [33]. The activation of iNKT cells together with α-galactosylceramide, in vivo, triggers a substantial effect on immune cells and changes in iron homeostasis, increasing its demand [34]. It may explain the negative correlation detected between iNKT lymphocytes and serum iron levels in end-stage CKD patients.

The co-culture of T lymphocytes and neutrophils demonstrated that neutrophils suppress proliferation of early but not late activated CD4^+^ and CD8^+^ cells. In our study, we found a negative correlation between neutrophils and T cells in DKD and nephropathy due to other causes in CKD patients [35]. T cells and serum albumin concentration positively correlate in end-stage CKD patients irrespective of disease etiology. Indeed, higher concentrations of serum albumin have been described at sites of infection, which activates a glycerol monolaurate suppressive effect on human T cells [36].

This study emphasizes the need to establish reference values for hematological and biochemical parameters to characterize the influence of etiological factors in CKD patients’ evolution to the end stage. Indeed, our group recently explored the differentiation bias of helper T cells in end-stage CKD patients, evidencing a reduced frequency of naïve T cells and FOXP3+ regulatory T cells, while effector memory T cells, Th1 cells, and Th17 cells are increased [37]. End-stage CKD patients with and without diabetes have no significant differences regarding the genetic expression of the transcription factors FOXP3, GATA3, RORC, and T-bet. However, T-bet and FOXP3 are significantly correlated in diabetic patients, while RORC correlates with FOXP3 in non-diabetics. Monitoring the relationship among leukocyte subpopulations and hematological and biochemical parameters in CKD patients can substantially enrich the information input into kidney failure risk platforms [38].

## 5. Conclusions

The flow cytometric characterization of leukocyte subpopulations in end-stage CKD patients unveils an increased frequency of inflammatory non-classical monocytes that positively correlates with total lymphocytes and B lymphocytes. When diabetes is the etiology of CKD, a significantly positive correlation occurs between pro-inflammatory monocytes and eosinophils, classical monocytes and neutrophils, and helper T cells and total monocytes. In DKD patients, B cells show a negative correlation with biochemical parameters such as alkaline phosphatase, parathyroid hormone, and ferritin, while iNKT cells negatively correlate with serum iron. However, in non-diabetic CKD patients, non-classical monocytes and total lymphocytes are significantly correlated, as well as T lymphocytes and serum albumin. Uremic environment differentially modifies leukocyte subpopulation, the serological concentration of biochemical parameters, and the correlation among these biomarkers in diabetic and non-diabetic end-stage CKD patients.

## Figures and Tables

**Table 1:** Fluorophore-conjugated monoclonal antibodies for flow cytometry assays.

		Catalog ID	Supplier
**Antibody-conjugated fluorophore**	**T lymphocytes**		
	PE Cy7 CD3	317334	BioLegend^®^
	APC CD4	ABIN 375816	antibodies-online Inc.
	PerCP Cy5.5 CD8	344710	BioLegend^®^
	PE CD45RA	304108	BioLegend^®^
	FITC CCR7	ABIN 674724	antibodies-online Inc.
	**B lymphocytes**		
	PercP Cy5.5 CD19	302230	BioLegend^®^
	**Monocytes**		
	PECy7 CD3	317334	BioLegend^®^
	FITC CD14	ABIN 93966	antibodies-online Inc.
	APC CD16	ABIN 2144226	antibodies-online Inc.
**Reagent**	Lysis Buffer	420301	BioLegend^®^

**Table 2:** Sociodemographic and etiopathogenic characteristics.

Variables	N = 20	% or IQR
**Gender**		
F	9	45%
M	11	55%
**Age (years)**	66.5 ± 16.5 *	(57–73.5)
**Duration of uremia (years)**		
(0–3)	4	20%
(4–6)	5	25%
(>7)	11	55%
**Etiology of end-stage CKD**		
Glomerulonephritis	3	15%
Hipertensive nephropathy	3	15%
Diabetes	11	55%
Chronic dysfunction (kidney graft)	2	10%
Renal lithiasis	1	5%
**Body mass index (BMI)**		
Underweight (<18.5)	1	5.6%
Normal (18.5–24.9)	10	55.6%
Overweight (25–29.9)	7	38.9%
**Hypertension**	11	55%
**Hepatitis C**	7	35%

M: male or F: female; * values expressed as median ± IQR (interquartile range) or absolute number (percentage of total cases).

**Table 3:** Analysis of hematological and biochemical parameters.

Parameters	N = 20	IQR	Reference Value ^a^
Hemoglobin (mg/dL)	11.3 ± 2.11	(10.6–12.7)	M: 13.5–17.5 mg/dL F: 12.0–16.0 mg/dL
Hematocrit (%)	35.5 ± 6.7	(33.2–39.9)	M: 41–53% F: 36–46%
Urea post-hemodialysis (mg/dL)	35.5 ±16	(26.5–42.5)	7–20 mg/dL
Creatinine post-hemodialysis (mg/dL)	2.6 ± 0.9	(2.1–3)	M: 0.7–1.3 mg/dL F: 0.6–1.1 mg/dL
TGP (U/L)	12 ± 10	(9–19)	7–56 U/L
TGO (U/L)	14 ± 6	(11–17)	5–40 U/L
Alkaline phosphatase (U/L)	140.5 ± 58	(122–180)	44–147 U/L
Total proteins (g/dL)	7.2 ± 0.8	(6.8–7.6)	6.0–8.3 g/dL
Albumin (g/dL)	3.8 ± 0.5	(3.7–4.2)	3.4–5.4 g/dL
CRP (mg/L)	1.2 ± 0.5	(0.9–1.4)	<1 mg/L
Serum calcium (mg/dL)	8.6 ± 0.8	(8.3–9.1)	8.5–10.2 mg/dL
Phosphorus (mg/dL)	5.2 ± 2.1	(4.6–6.7)	2.4–4.1 mg/dL
Iron (μM/L)	10.2 ± 8.2	(8.4–16.6)	10.74–30.4 μM/L
Transferrin (mg/dL)	180 ± 51	(153.5–204.5)	M: 215–360 mg/dL F: 245–370 mg/dL
Kt/v	1.7 ± 0.3	(1.5–1.8)	≥1

Median ± IQR (interquartile range); TGP: glutamate pyruvic transaminase; TGO: oxalacetic transaminase; CRP: C-reactive protein; Kt/v: relationship between the amount of urea eliminated in a session (Kt) and the volume of distribution of urea in the patient (V). ^a^ Laboratory of Edgardo Rebagliati Martins National Hospital.

**Table 4:** Quantification of leukocyte subpopulations in end-stage CKD patients.

Cells	Reference Values ^a^	N = 20	IQR
Leukocytes (cells/mm^3^)	4500–10,000	7190 ± 1905	6310–8215
Total monocytes (cells/mm^3^)	500–1200	536.1 ± 221.4	471.2–692.6
Neutrophils (cells/mm^3^)	1800–7000	4.5859 ± 1277.9	3979.2–5257.2
Total lymphocytes (cells/mm^3^)	1000–4800	1463.1 ± 732.5	1172.9–1905.5
T CD3^+^ (cell/mm^3^)	690–2540	1195.6 ± 570.6	837.3–1407.8
T CD3^+^CD4^+^ (cells/mm^3^)	410–1590	587.4 ± 498	395.6–893.6
T CD3^+^CD8^+^ (cells/mm^3^)	190–1140	417.8 ± 238.1	293.5–531.5
B CD19^+^ (cells/mm^3^)	90–660	91.8 ± 122.7	48.9–171.7
NK CD16^+^CD56^+^ (cells/mm^3^)	90–590	289.9 ± 178.6	217.7–396.3
iNKT CD3^+^CD16^+^CD56^+^ (cells/mm^3^)	NA	0.29 ± 0.80	0.15–0.95
T CD4^+^/CD8^+^ ratio	≥2.0	1.4 ± 0.79	1.1–1.9

Median ± IQR (interquartile range); ^a^ Laboratory of Edgardo Rebagliati Martins National Hospital. NA: No reference values information is available at the cited laboratory.

**Table 5:** Distribution of monocyte subpopulations in end-stage CKD patients.

Subpopulation of Monocytes	Reference Values ^a^	N = 20	IQR
Classical monocytes CD14^++^CD16^−^ (cell/mm^3^)		329.7 ± 232.6	(248.5–481.1)
%	80–95%	69.4 ± 19.3	(55–74.3)
Intermediate monocytes CD14^+^CD16^+^ (cell/mm^3^)		35.3 ± 43.1	(24.03–67.1)
%	2–11%	7.7 ± 5	(5.2–10.2)
Non-classical monocytes CD14^+^CD16^++^ (cell/mm^3^)		129.5 ± 115.8	(84.2–199.9)
%	2–8%	21.8 ± 15.95	(17.1–33)

Median ± IQR (interquartile range) or mean ± SD (standard deviation); ^a^ reference values taken from BD Biosciences.

**Table 6:** Correlation between leukocyte subpopulations and biochemical parameters.

Variables		DKD (n = 11)		Nephropathy Due to Other Causes (n = 9)	
		* Rho	*p* Value	* Rho	*p* Value
T lymphocytes	Serum albumin (g/dL)	+0.167	0.623	+0.874	**0.005**
T lymphocytes	Neutrophils (cells/mm^3^)	−0.118	0.729	−0.667	**0.049**
NK CD3^−^CD16^+^CD56^+^ (cells/mm^3^)	Serum iron (g/dL)	−0.564	0.071	−0.214	0.645
iNKT CD3^+^CD16^+^CD56^+^ (cells/mm^3^)	Serum iron (g/dL)	−0.719	0.029	−0.342	0.452
B CD19^+^ (cells/mm^3^)	Alkaline phosphatase (U/L)	−0.764	0.006	+0.179	0.702
B CD19^+^ (cells/mm^3^)	Parathyroid hormone	−0.929	0.003	0.200	0.747
B CD19^+^ (cells/mm^3^)	Ferritin (mg/dL)	−0.893	0.007	−0.700	0.188
T CD3^+^CD4^+^	Serum albumin (g/dL)	0.033	0.925	+0.934	**0.001**
T CD3^+^CD4^+^	Parathyroid hormone	−0.929	0.003	−0.100	0.873
T CD3^+^CD4^+^	Neutrophils (10^3^ cells/mm^3^)	+0.127	0.709	−0.683	**0.042**
T CD3^+^CD4^+^	Total monocytes	+0.627	0.039	−0.017	0.966

* Spearman correlation test. DKD: diabetic kidney disease; significant *p* values (*p* ≤ 0.05) are presented in bold.

**Table 7:** Correlation between non-classical monocytes and lymphocytes in end-stage CKD patients.

Variables		Spearman’s Rho	*p* Value
Non-classical monocyte	Total lymphocytes	+0.495	**0.027**
Non-classical monocyte	B lymphocytes	+0.567	**0.009**

Significant *p* values (*p* ≤ 0.05) are presented in bold.

**Table 8:** Correlation between monocyte and leukocyte populations in DKD and nephropathy due to other causes in end-stage CKD patients.

Variables		DKD (n = 11)		Nephrophaty Due to Other Causes (n = 9)	
		Rho	*p*-Value	Rho	*p*-Value
Classic monocyte	Neutrophils	+0.627	**0.039**	+ 0.700	**0.036**
Non-classical monocyte	Eosinophils	+0.691	**0.019**	−0.2667	0.488
Non-classical monocyte	B lymphocytes	+0.591	0.056	+0.500	0.171
Non-classical monocyte	Total lymphocytes	+0.309	0.355	+ 0.683	**0.042**

Significant *p* values (*p* ≤ 0.05) are presented in bold.

## Data Availability

The original contributions presented in this study are included in this article; further inquiries can be directed to the corresponding author/s.
